# NOX family NADPH oxidases in mammals: Evolutionary conservation and isoform-defining sequences

**DOI:** 10.1016/j.redox.2023.102851

**Published:** 2023-08-12

**Authors:** Bahareh Nazari, Vincent Jaquet, Karl-Heinz Krause

**Affiliations:** a Department of Pathology & Immunology, Faculty of Medicine, University of Geneva, Geneva, Switzerland; b READS Unit, Faculty of Medicine, University of Geneva, Geneva, Switzerland

**Keywords:** Reactive oxygen species, Orthologs, Evolution, Multiple alignment

## Abstract

NADPH oxidases are superoxide-producing enzymes that play a role in host defense, biosynthetic pathways, as well as cellular signaling. Humans have 7 NOX isoforms (NOX1-5, DUOX1,2), while mice and rats lack NOX5 and therefore have only 6 NOX isoforms. Whether all human NOX isoforms or their subunits (CYBA, NCF1, 2, 4, NOXO1, NOXA1, DUOXA1, 2) are present and conserved in other mammalian species is unknown. In this study, we have analyzed the conservation of the NOX family during mammalian evolution using an in-silico approach. Complete genomic sequences of 164 mammalian species were available. The possible absence of genes coding for NOX isoforms was investigated using the NCBI orthologs database followed by manual curation. Conservation of a given NOX isoform during mammalian evolution was evaluated by multiple alignment and identification of highly conserved sequences. There was no convincing evidence for the absence of NOX2, 3, 4, and DUOX1, 2 in all the available mammalian genome. However, NOX5 was absent in 27 of 31 rodent, in 2 of 3 lagomorph and in 2 out of 18 bat species. NOX1 was absent in all sequenced Afrotheria and Monotremata species, as well as in 3 of 18 bat species. NOXA1 was absent in all Afrotheria and in 3 out of 4 Eulipotyphla species. We also investigated amino acid sequence conservation among given NOX isoforms. Highly conserved sequences were observed for most isoforms except for NOX5. Interestingly, the highly conserved region of NOX2 sequence was relatively small (11 amino acids), as compared to NOX1, 3, 4.

The highly conserved domains are different from one NOX isoform to the other, raising the possibility of distinct evolutionary conserved functional domains. Our results shed a new light on the essentiality of different NOX isoforms. We also identified isoform-defining sequences, i.e., hitherto undescribed conserved domains within specific NOX isoforms.

## Highlights

• Comparison of Mammalian NOX orthologues highlighted highly variable degree of conservation between NOX isoforms and subunits.
• NOX5 sequence is the least conserved while DUOX2 is the most conserved.
• NOX1 was absent from 11 mammalian species including Monotremata and Afrotheria.
• NOX5 was absent in 37 species, including most rodents, except marmots and squirrels.

## 1 Introduction

Nicotinamide adenine dinucleotide phosphate (NADPH) oxidases (NOX) are a group of transmembrane enzymes that catalyze electron transport reactions across cellular membranes. The outcome of these reactions is the production of superoxide radical anions and hydrogen peroxide. During this process, cytosolic NADPH acts as electron donor while extra cytosolic oxygen is the electron acceptor [[1], [2], [3]]. The human NOX family comprises seven isoforms including NOX1 to NOX5, DUOX1, and DUOX2. NOX isoforms can be classified by their tissue distribution and mechanism of action. Their activation process can be convoluted; some isoforms need additional subunits to create a fully functional enzyme. For example, NOX1-3 require cytosolic subunits NCF1, NCF2, NCF4, NOX organizer type 1(NOXO1), NOX activator type 1(NOXA1); CYBA-a transmembrane subunit-is needed for the function of NOX1-4; intracellular Ca^2+^ binding and phosphorylation induce conformational changes and enzyme activation in NOX5, DUOX1, and DUOX2; DUOX1 and DUOX2 need the maturation factors DUOXA1 and DUOXA2 to function [2,4,5].

Despite heterogeneity between NOX isoforms, their catalytic core is composed of a transmembrane domain and a cytosolic dehydrogenase domain. Indeed, six membrane-spanning domains (seven in DUOX1 and DUOX2), two-paired heme binding sites, and C-terminus located NADPH and flavin adenine dinucleotide (FAD) binding sites are common among all NOX. These conserved structures in the members of the NOX family explain their common enzymatic functions [6].

Initially, the NOX family was observed and described only in eukaryotic organisms. Yet, studies within the last decade have demonstrated the existence of prokaryotic NOX that possess a catalytic core resembling that of eukaryotic NOX. However, these prokaryotic NOX sequences are constitutively active as single polypeptide chains, without needing cytosolic factors or an activation process (such as the NOX found in *Streptococcus pneumoniae*). Therefore, prokaryotic NOX differs significantly from their eukaryotic counterparts [7,8]. The NOX family emerged in eukaryotic organisms during the early stages of evolution. Phylogenic analysis suggests the presence of an ancestral NOX whose function was regulated by Ca2+ through the presence of EF-hand domains in the cytosolic N-terminus. The N-terminus was lost with the emergence of NOX1-4 isoforms due to gene duplication. Regulation of these isoforms required the development of separate regulatory subunits, which appeared concomitantly with the disappearance of the EF-hand containing N-terminus [9].

A multiple sequence alignment serves as a molecular fossil. By examining the patterns of amino acid variations among aligned sequences, we gain invaluable insights into the evolutionary pressures that have shaped them over millions of years. Detecting conserved amino acids through multiple sequence alignment is particularly useful. These conserved residues often hold significant importance in the protein's function or structure, and any mutations occurring in these specific residues can have a harmful impact on the protein's functionality [10,11].

All seven NOX isoforms NOX1-5 and DUOX1-2 are generally assumed to be present in mammals. However, there is no study providing a thorough evaluation of this notion. Here we investigated the distribution of NOX isoforms in 164 mammalian species for which there is a complete genome sequence available in the ortholog database. Correspondingly, we showed conserved sequences of each isoform among mammalian species.

## 2 Methods

### 2.1 Database searches

Mammalian genome sequences in the NCBI ortholog database were searched for each NOX isoforms (latest update was in November 2022). In a first step, the presence of NOX isoforms was evaluated using this database. When the ortholog database provided no or ambiguous information, the presence of NOX isoforms in the species was further assessed as follows. We visualized the neighboring genes of the respective NOX genes (using genome viewer: https://www.ncbi.nlm.nih.gov/genome/gdv/and NCBI Gene database: https://www.ncbi.nlm.nih.gov/gene/) (Supplementary Fig. 1).

The following situations were encountered (supplementary excel file):

1. The neighboring genes were present and there were no obvious sequencing gaps, yet there was no sign of the respective NOX gene. In this case we considered the NOX isoform to be absent.
2. The neighboring gene sequences were also missing. In this case, we concluded that the region was not sequenced, and we did not consider the respective NOX isoform as absent.
3. The neighboring gene sequences were present, but there were sequencing gaps between the neighboring genes. In this case, we concluded that the region was poorly sequenced, and we did not consider the respective NOX isoform as absent.
4. The neighboring genes were present with an open reading frame between them. In this case, we blasted the open reading frame. In most cases, the open reading frame turned out to be a NOX homologue that had not been identified in the NCBI ortholog database. In this situation, we considered the NOX gene to be present.

### 2.2 Identification of conserved ortholog sequences

In order to compare amino acid sequences of each NOX isoform in mammalian species and find possible conservation, constraint-based multiple alignment tool (COBALT) of NCBI was used (https://www.ncbi.nlm.nih.gov/tools/cobalt/cobalt.cgi?LINK_LOC=BlastHomeLink). COBALT generates pairwise constraints from conserved domain databases, protein motif databases, and sequence similarity. For this purpose, the tool utilizes RPS-BLAST, BLASTP, and PHI-BLAST. Then, COBALT incorporates pairwise constraints achieved from different sources into a progressive multiple alignment. The results are used by algorithms to generate a guide tree which conducts the actual alignment process. Finally, the refinement step improves the multiple alignment results [12]. In this analysis, Cobalt's default parameters were used. (Alignment parameters: gap opening penalties and extensions: −11 and −1, respectively; end-gap penalties opening and extension: −5 and −1, respectively) (CDD parameters: use RPS BLAST: on; Blast E-value: 0.005, Find conserved columns and recompute: on) (query clustering parameters: use query clusters: on; word size: 4; max cluster distance: 0.8; alphabet: SE-B15)

The output results are color coded, and coloring is only applied to alignment positions which do not have any gaps. Based on the relative entropy threshold of the residue, amino acids are detected as highly conserved (red) or less conserved (blue). Non-conserved ones are indicated as gray.

## 3 Results

### 3.1 Absence of NOX isoforms in mammals

Mammals are generally considered to have 7 NOX isoforms: NOX1-5 and DUOX1-2. However, this notion has not been rigorously investigated. To address this question, we have searched for orthologs of the 7 human NOX in 164 mammalian species, for which a complete genome sequence is available. Our search strategy is described in the method section. Absence of a given NOX isoform was considered as a relevant result and in the case of doubt (i.e., poor sequencing of a given genomic region), the respective NOX isoform was not considered as absent.

To report a NOX isoform as absent, the following criteria should be met:

- absence in the NCBI's ortholog database
- manual verification of sequence absence as described in method section
- lack of a NOX open reading frames in proximity of neighboring genes

The panel on the left side of Fig. 1 shows a phylogenetic tree of mammals [13]. Mammals are considered as a class of animals, consisting of 3 subclasses. Two subclasses are non-placental animals: i) the egg-laying Monotremata and ii) Marsupialia, that raise the immature offspring in a pouch. The third subclass, Placentalia, corresponds to placental mammals, which are by far the largest subclass.

**Fig. 1:**
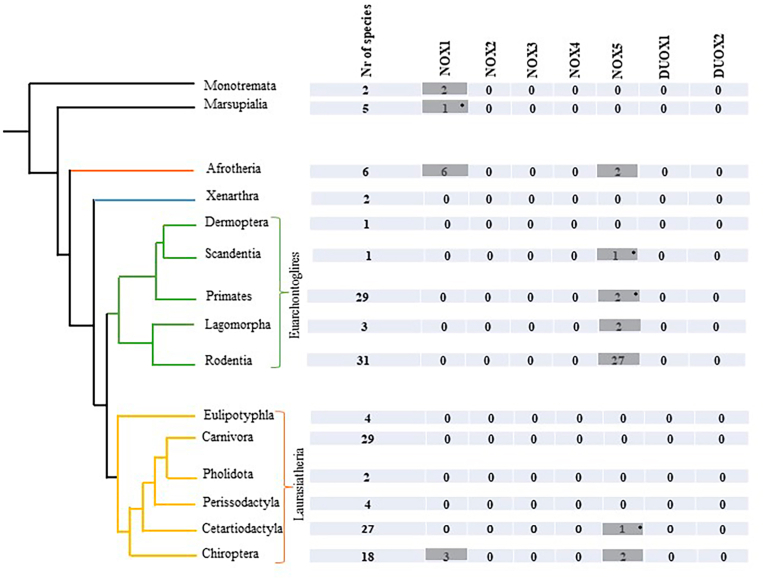
Diagram showing the distribution of species lacking a given NOX isoform across mammalian's phylogenetic tree. The first column of table shows the number of fully sequenced species within each group. In the following columns, the numbers refer to species without the specific NOX isoform. A value of zero indicates that all species in the corresponding group have that NOX isoform. Gray highlight indicates the absence of a specific NOX isoform in the given clade or order. * indicates a singlet species lacking NOX isoform without known close evolutionary relatives. Mammalian's phylogenetic tree was adapted from Ref. [13].

Two complete genome sequences are available for Monotremata: *Ornithorhynchus anatinus* (platypus) and *Tachyglossus aculeatus* (echidna), as well as 5 from Marsupialia. The remaining 157 sequences belong to placental mammals. Placental mammals are divided into four major clades: Afrotheria (n = 6), Xenarthra (n = 2), Euarchontoglires (n = 65), and Laurasiatheria (n = 84). Afrotheria and Xenarthra developed under confined conditions of isolated continents. Indeed Africa (Afrotheria) and South America (Xenarthra) were island continents for prolonged periods [14,15]. The best-known examples of Afrotheria are *Loxodonta africana* (elephant) and *Trichechus manatus* (manatee) as well as *Mammuthus* (the extinct mammoths). The best-known examples of Xenarthra are *Choloepus didactylus* (sloth) and *Dasypus novemcinctus* (armadillo). The other two clades Euarchontoglires and Laurasiatheria have mostly evolved in Europe and Asia. These clades are divided in orders which themselves consist of multiple species.

Fig. 1 provides the information on species lacking a given NOX isoform. The first column shows the number of fully sequenced species within the different clades or orders. The numbers in the following columns refer to species without the respective NOX isoform. Zero means that all species in the respective order either have that NOX isoform or lack convincing evidence for its absence. A total of 49 species showed the absence of one NOX isoform: 12 species were lacking NOX1, while 37 species were lacking NOX5. In contrast, NOX2, NOX3, and NOX4 were present in all mammalian species. When the genomic regions of the respective NOX isoforms were poorly sequenced, we did not conclude the absence of a given NOX. For example, we could not find NOX4 in the *Meriones unguiculatus* (Mongolian gerbil) in the NCBI's ortholog database. However, a detailed inspection of the respective genomic region revealed that NOX4 corresponding region on the genome was not available (presence of gaps). Therefore, we did not conclude that NOX4 is absent in the Mongolian gerbil. Similarly, for most mammalian species, DUOX1 and DUOX2 were found in the ortholog database. In a few remaining cases, these isoforms were detected by inspection of the respective genomic sequences and their neighboring gene using the genome browser. For example, an ortholog database search did not find DUOX2 in the *Acinonyx jubatus* (cheetah) genome. However, the inspection of the genomic region around the neighboring genes allowed to detect a DUOX2 sequence. Thus, there is no evidence for mammalian species that lack NOX2, NOX3, NOX4, DUOX1 or DUOX2. On the other hand, there is clear evidence for mammalian species lacking NOX1 and/or NOX5.

Fig. 2 shows the number of species in each group that lack a particular subunit of NOX in mammals (organization of Fig. 2 is the same as Fig. 1). There is no evidence for mammalian species lacking NCF1, NCF2, NCF4, or CYBA. A total of 11 species were found to lack NOXA1, and/or NOXO1. We also found one species lacking DUOXA1 and DUOXA2.

**Fig. 2:**
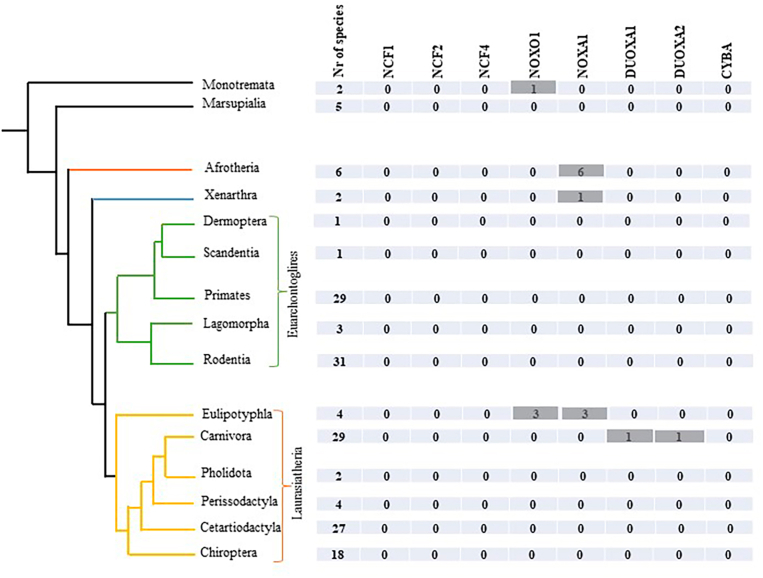
Diagram showing the distribution of species lacking a given NOX subunit across mammalian's phylogenetic tree. The first column of table shows the number of fully sequenced species within each group. In the following columns, the numbers refer to species without the specific NOX subunit. A value of zero indicates that all species in the corresponding group have that NOX subunit. Gray highlight indicates the absence of a specific NOX subunit in the different groups of mammals. Mammalian's phylogenetic tree was adapted from Ref. [13].

Our investigation showed that NOX1 was present in 152 mammalian species, while 12 species were likely to lack this isoform. This finding was based on retrieval from the ortholog database, as well as detailed inspection of the respective chromosomal regions in the NCBI genome viewer. Most importantly, however, the absence of NOX1 in certain species is confirmed by evolutionary proximity. Indeed, NOX1 is absent in both sequences available from the mammalian subclass Monotremata. Within the subclass of Placentalia, NOX1 was absent in all sequences from Afrotheria. A third example would be within the order of Chiroptera(=bats) which belongs to the clade of Laurasiatheria within the subclass placental mammals. Indeed, in three closely related bat species *Artibeus jamaicensis*, *Sturnira hondurensis* and *Phyllostomus discolor*, we did not detect NOX1. There were singlet species that might not have NOX1 (*Monodelphis domestica*) (Supplementary Table 1), However there was no confirmation by an evolutionary closely related species.

The absence of NOX5 in mice and rats is well documented [16]. From this observation, it has been extrapolated that NOX5 was absent in all rodents [17]. Similarly, NOX5 was found in order of Lagomorpha (=rabbits) and it has been extrapolated that NOX5 is present in lagomorphs [18]. However, based on our analysis, the situation is more complex. NOX5 is absent in 37 out of 164 mammalian species. Thus, it is absent in almost 22% of mammalian species, which makes NOX5 the most “dispensible” isoform within the NOX family. Out of the 31 rodent species with an available genome sequence, 27 are lacking NOX5. However, available sequence from the closely related marmots (*Marmota flaviventris* and *Marmota marmota*), and squirrels (*Ictidomys tridecemlineatus Urocitellus parryii*) show the presence of NOX5. Inversely, in Lagomorpha, which are subdivided into Leporidae (rabbits) and Ochotonidae (pikas), we found NOX5 only in *Oryctolagus cuniculus* (= European rabbit), while NOX5 was absent in *Ochotona curzoniae* and *Ochotona princeps* (pika species). NOX5 was also absent in two closely related species of the Afrotheria superorder (*Echinops telfairi* and *Elephantulus edwardii*), as well as in two closely related Chiroptera species (*Rhinolophus ferrumequinum* and *Hipposideros armiger*). There were also four species apparently lacking NOX5, but they were singlets without supporting evidence from another closely related species*: Tupaia chinensis*, *Monodon monoceros*, *Otolemur garnettii*, *Saimiri boliviensis* (Supplementary Table 1).

NOXO1 and NOXA1 are known subunits of NOX1 [19] and NOX3 [20,21]. The NOXA1 subunit was absent in 10 out of 164 mammalian species. All 6 species in the superorder Afrotheria are lacking NOXA1 in addition to lacking isoform NOX1. There are also other four placental mammals that do not appear to possess NOXA1: *Sorex araneus*, *Talpa occidentalis*, *Condylura cristata* (three closely related Eulipotyphla species) and *Choloepus didactylus* (Xenarthra superorder). In these placental mammals there is no concurring loss of NOX1.

The NOXO1 subunit is absent in 4 out of 164 mammalian species, namely the closely related Eulipotyphla species *Sorex araneus*, *Talpa occidentalis*, *Condylura cristata* which also lack NOXA1 (see above). It also appears that NOXO1 is missing in the Monotremata species *Ornithorhynchus anatinus.*

A search of the ortholog database together with a separate inspection of the NCBI genome viewer showed that DUOXA1 and DUOXA2 maturation factors were present in all mammals except for the *Ailuropoda melanoleuca* (giant panda). In this species, the neighboring genes were present and there were no sequencing gaps, but still, there was no sign of the respective DUOXA1 and DUOXA2. In addition, a blast search of unnamed open reading frames in the corresponding chromosomal region did not result in the identification of these maturation factors. Based on this evidence, we considered the possibility that the DUOXA1 and DUOXA2 subunits were absent in the giant panda.

### 3.2 Conservation of sequences within NOX isoforms in mammals

So far, we have investigated the presence or absence of given NOX isoforms and subunits in different mammalian species. In the following part of our investigations, we have looked for sequence conservation and variations of the different NOX isoforms and subunits in all mammals available in the ortholog database. For this purpose, we used the COBALT multiple alignment tool. This tool uses blasts (RPS-BLAST, BLASTP, and PHI-BLAST) to find pairwise constraints and then, progressive multiple alignments are performed on these pairwise constraints. The output is presented with the following color-code: red indicates highly conserved amino acids while blue highlights represent less conserved areas. The gray parts indicate non-conserved parts in which there is no gap. The parts of the sequence that are shown as a horizontal red line indicate amino acid gaps. It is worth noting that performing multiple sequence alignments of closely related species, such as primates, often highlighted numerous highly conserved amino acids. However, the COBALT output shown here exclusively illustrates the highly conserved amino acids, which are common among all mammalian species.

Fig. 3 represents an overview of multiple alignment of each NOX isoform in all mammals available in the ortholog database. The position of functionally relevant domains is indicated below the alignments (PHD = peroxidase homology domain; CBD= Ca2+-binding domain; TM = transmembrane domain; DH = dehydrogenase domain). A visual inspection of the alignments shows the highest degree of conservation in DUOX2 and in NOX3. In contrast, NOX5 is the least conserved isoform across mammals. The situation is intermediate for NOX1, NOX2, NOX4 and DUOX1, however with fewer highly conserved sequences as compared to DUOX2 and NOX3.

**Fig. 3:**
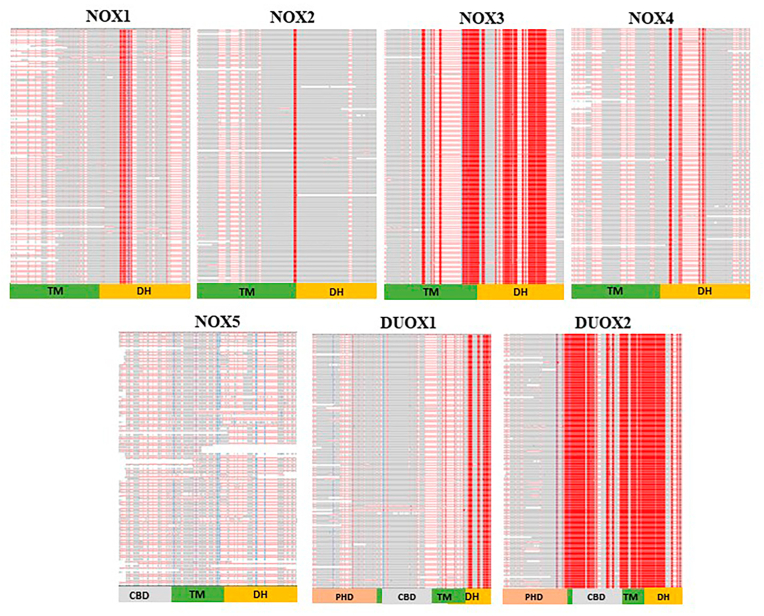
Overview of amino acid sequence alignment of different NOX isoforms across mammals. This figure summarizes the conservation degree of each NOX isoform throughout mammals. The red color indicates highly conserved amino acids while the gray color demonstrates not conserved amino acids. The bottom block shows the location of the different domain for each NOX isoform. TM: transmembrane domain; DH: dehydrogenase domain; CBD: Ca^2+^ binding domain; PHD: peroxidase homology domain. (For interpretation of the references to color in this figure legend, the reader is referred to the Web version of this article.)

We next investigated the size and localization of the conserved sequences in the different NOX isoforms (Fig. 4; amino acids of conserved sequences are also shown in Supplementary Figs. 2–8). The number of highly conserved amino acids for the isoforms are as follows: 36 out of 564 aa (6%) for NOX1, 11 out of 570 aa (2%) for NOX2, 253 out of 568 aa (45%) for NOX3, 36 out of 578 aa (6%) for NOX4, 182 out of 1551 aa (12%) for DUOX1, 815 out of 1548 aa (53%) for DUOX2. As discussed above, there is no amino acid which is highly conserved throughout the mammalian NOX5 sequences.

**Fig. 4:**
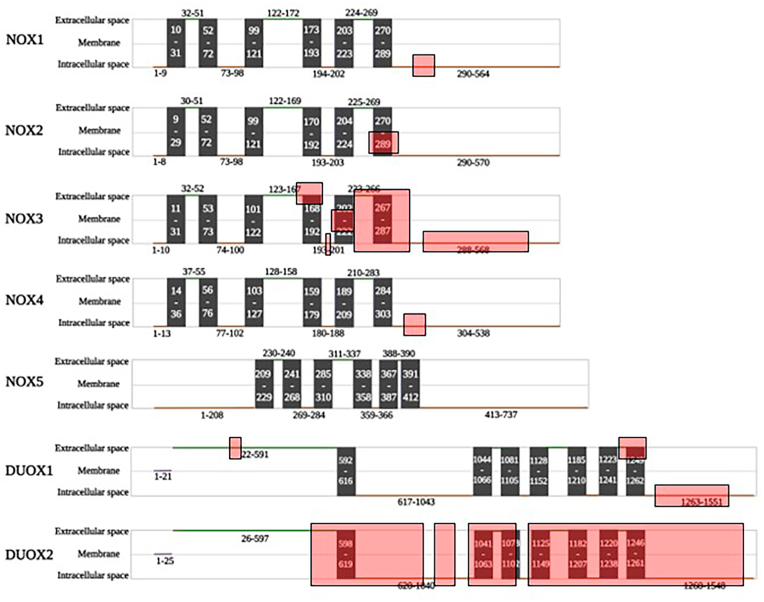
Scheme of human NOX isoforms' structure. The red highlights show the conserved parts found in all mammals. (For interpretation of the references to color in this figure legend, the reader is referred to the Web version of this article.)

The relevant functional domains found in all NOX are: i) 6/7 transmembrane domains, ii) a dehydrogenase domain consisting of FAD and NADPH binding sites. The topology of the conserved regions within the different NOX isoforms are as follows (amino acid numbers are given with reference to the human isoform):

NOX1: 36 out of 564 aa in NOX1 are conserved among mammals. The conserved amino acids (aa) are all in the dehydrogenase domain. Some conserved aa are found in the FAD binding site (as shown in Supplementary Fig. 2). Indeed, 19 out of 23 amino acids in FAD binding site are highly conserved. On the other hand, the NADPH binding site of NOX1 does not have highly conserved amino acid sequences.

NOX2: The conserved part of NOX2 is quite small; with only 11 out of 570 being conserved. The sequence of these conserved amino acids is “MFLYLCERLVR”, present in the 6th transmembrane domain from positions 277 to 287. Neither the FAD binding sites nor the NADPH binding sites of NOX2 are highly conserved among mammals, as shown in Supplementary Fig. 3.

NOX3: A high number of amino acids in NOX3 is highly conserved among mammals (253 out of 568). Strikingly, there is a complete conservation of loop E as well as the entire transmembrane domain 6. In addition, conserved parts are found within loop C, loop D, transmembrane domains 4 and 5. In the dehydrogenase domain, the majority of NADPH binding site is completely conserved, while there is not strong conservation within the FAD binding site (Supplementary Fig. 4).

NOX4: The conserved part of NOX4 is small; 36 out of 578 aa are conserved across mammals. They are present in the dehydrogenase domain. While there are some conserved aa in FAD binding site of NOX4, NADPH binding site of this isoform is not highly conserved (Supplementary Fig. 5).

DUOX1: 182 out of 1551 aa in DUOX1 are conserved among mammals. These conserved amino acids are present in peroxidase homology domain, the entire loop E, transmembrane domain 7 and the dehydrogenase domain. The NADPH binding site is partially conserved, while the FAD binding site is completely conserved (Supplementary Fig. 7).

DUOX2: This isoform has the highest degree of conservation compared to other NOX isoforms. Over half of DUOX2 sequence- 815 out of 1548 aa-is conserved across mammals. These conserved amino acids are distributed throughout the sequence, with all transmembrane domains 1–2 and 4–7 fully conserved and 80% of transmembrane domain 3 (20 out of 25 amino acids) conserved. The NADPH binding site is partially conserved, while the FAD binding site is completely conserved (Supplementary Fig. 8).

Fig. 5 represents a summary of the multiple alignments of each NOX subunit among all mammals found in the ortholog database. Visually, NCF2 displays the highest level of conservation among all the subunits. DUOXA2 and DUOXA1 show a relatively high level of conservation. Conversely, NOXA1 and NOXO1 seem to be the subunits with the least conservation across the mammalian species. For NCF1, NCF4, and CYBA, the conservation of amino acids is intermediate, with a lesser number of conserved amino acids in comparison to NCF2, DUOXA1, and DUOXA2.

**Fig. 5:**
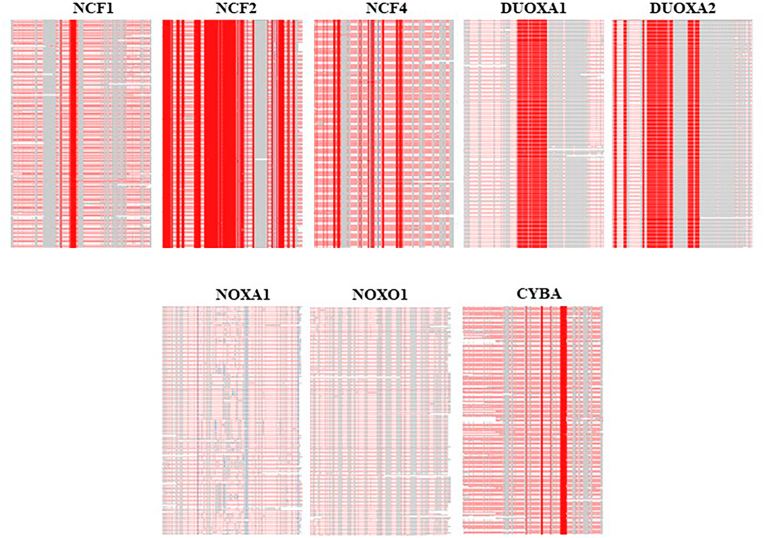
Overview of amino acid sequence alignment of different NOX subunits across mammals. This figure summarizes the conservation degree of each NOX subunit throughout mammals. The red color indicates highly conserved amino acids while the gray color demonstrates not conserved amino acids. (For interpretation of the references to color in this figure legend, the reader is referred to the Web version of this article.)

We then examined the location of the conserved sequences in the different NOX subunits. The number of conserved amino acids for each subunit is as follows: 44/390 aa (11%) in NCF1, 385/526 aa (73%) in NCF2, 105/339 aa (31%) in NCF4, 110/483 aa (23%) in DUOXA1, 124/320 aa (39%) in DUOXA2, and 38/195 aa (19%) in CYBA. As mentioned earlier, there is no conserved amino acid throughout the mammalian tree NOXA1 and NOXO1 sequences.

The topology of the conserved regions within the different NOX subunit is as follows:

NCF1: Out of 390 aa in NCF1, 44 are conserved across mammals. 42 of the conserved aa are detected in the first Src homology 3 (SH3) domain, with 2/3 of the domain being conserved. The other 2 conserved aa located in proximity of the first SH3 domain, in the sequence between phox (PX) and SH3 domains (aa 148 and 150). Despite the relatively high sequence identity between NOXO1 and p47phox (∼25%) and a high degree of similarity of the functional domains, there are no conserved aa in NOXO1 subunit.

NCF2: This subunit shows the highest degree of conservation among mammals compared to other NOX subunits. Two thirds of the NCF2 sequence (i.e., 385 out of 526 amino acids) is conserved across all mammals. Almost all tetratricopeptide repeat (TPR) domain, all activation domain (AD), and the first SH3 domain are conserved. Additionally, two-thirds of the sequence between the first SH3 domain and Phox and Bem 1 (PB1) domain is conserved. There are no conserved amino acids in the PB1 domain, making it the least conserved region of NCF2. Nearly half of the sequence between the PB1 domain and the second SH3 domain is conserved, and a large part of the second SH3 domain is conserved. Although NOXA1 and NCF2 share nearly 28% sequence identity and a high degree of domain structure similarity, there are no conserved amino acids in the NOXA1 subunit.

NCF4: Out of 339 amino acids in NCF4, 105 are conserved across all mammals. The conserved aa are distributed in the N terminal of protein, PX domain and 2/3 of SH3 domain.

DUOXA1: Out of 483 aa in DUOXA1, 110 are conserved among mammals. The conserved amino acids are in the entire extracellular domain and nearly a quarter of the third transmembrane domain.

DUOXA2: Out of 320 aa in DUOXA2, 124 are conserved in mammals, mainly in transmembrane domains 1, 2 (the whole domain), and 3 as well as topological domains 1, 2 (the whole domain), and 3. Cysteine 167, crucial for a disulfide bond with DUOX2, is conserved in all mammals.

CYBA: Out of a total of 195 aa in the CYBA subunit, 38 aa are conserved throughout mammalian species. They are distributed within the N terminal and the transmembrane domains of the protein.

## 4 Discussion

In this study we have analyzed NOX sequences in mammalian species with a specific focus on two questions: i) loss of isoforms, and ii) highly conserved regions within isoforms. We did not find any evidence for acquisition of novel NOX isoforms during mammalian evolution, however in some species certain isoforms were lost. Loss of isoforms was typically observed in evolutionary related species and at least in some instances, isoform-typical subunits were lost concomitantly. When analyzing highly conserved protein sequences, it was striking to observe that the highest conserved regions were very distinct between different isoforms. For example, while the Ca2+-binding domain of DUOX2 shows a high degree of conservation, this is not the case for the Ca2+-binding domain of DUOX1.

The NOX family of enzymes appeared early during evolution and is present in prokaryotes and eukaryotes. Most mammals have 7 different NOX isoforms, NOX1-NOX5 as well as DUOX1-DUOX2. In this study, we conducted a comprehensive analysis of these 7 NOX isoforms across 164 different mammalian species with complete genome sequences available in the orthologs database. Our results revealed that NOX2-4 and DUOX1, 2 are present in all mammalian species. However, NOX1 was absent in 12 species and NOX5 was absent in 37 species. Interestingly, most of the species that lacked either NOX1 or NOX5 were evolutionarily related.

The absence of NOX1 and NOX5 in certain species is unlikely to be explained by a selective acquisition of these isoforms during mammalian evolution. Indeed, NOX5 and NOX1-like enzymes are found in several non-mammalian species. It rather appears that – in some instances - NOX5 and NOX1 were lost by specific mammalian species. This loss appears to be closely related to the evolution of a given mammalian order. Strikingly the absence of NOX5 is common in rodents and in lagomorphs (27 out of 31 rodent species and 2 out of 3 lagomorph species do not have NOX5). Thus, NOX5 has most likely lost its physiological importance in these orders. As NOX5 is thought to be important in sperm capacitation [22], the lack of NOX5 might point towards a different sperm biochemistry in rodents and lagomorphs. Even more striking is the absence of NOX1 and NOXA1 in the clade of Afrotheria. Mammals of this clade are thought to have evolved in isolation after the separation of Africa from other continents. The concomitant loss of NOX1 and its cytoplasmic subunit NOXA1 strongly suggests that during the isolated evolution of Afrotheria, this isoform lost its essential function. Interestingly, there is a second cytoplasmic subunit of importance for the function of NOX1, namely NOXO1. So, why did this subunit persist despite the loss of an essential function of NOX1? The answer most likely lies in the fact that NOXO1 is essential for the function of the NOX3 isoform [2], which plays a key role in formation of otoconia and hence the sense of equilibrium [23]. Indeed NOXO1-deficient mice have significant equilibrium problems (*head slant* mice) [20]. The NOX1 isoform also is absent in Monotremata species (platypus and echidna); yet in Monotremata NOXA1 is present, raising the possibility that NOXA1 has a NOX1-independent function in Monotremata, but not in Afrotheria.

A puzzling observation is the fact that 3 out of 4 species of the order Eulipotyphla have lost the NOXO1 and NOXA1 subunits, without having lost any of the NOX isoforms. One might speculate that in this order the closely related NCF1 and NCF2 subunits are functioning as substitutes. However further research will be necessary to test this hypothesis.

Analysis of amino acid conservation among mammalian species yielded astonishing results. It is often assumed that the functionally well-defined regions of NOX, in particular the dehydrogenase domain with its NADPH and FAD binding sites, are highly conserved. Yet, our analysis showed that only NOX3 and DUOX2 have extended regions of highly conserved sequences in the dehydrogenase domain among mammals. There are also some smaller conserved dehydrogenase regions in NOX1, NOX4, and DUOX1 (6%, 6%, 12% respectively), while there are almost no such regions in NOX2 and NOX5. Concerning transmembrane regions, there are i) extended stretches of high conservation in DUOX2; ii) some short stretches of high conservation in NOX2, NOX3, and DUOX1; and iii) no regions of high conservation in NOX1, NOX4, and NOX5. With respect to the Ca2+-binding domains (present in NOX5, DUOX1, and DUOX2), there are long stretches of conservation in DUOX2, a short stretch in DUOX1, and no conservation in NOX5. Finally, there were short stretches of high conservation within the peroxidase homology domains of both DUOX isoforms.

Amino acid conservation of NOX subunits during mammalian evolution is variable as seen for NOX isoforms. However, there is only a partial correlation between the conservation of the NOX isoforms and the corresponding NOX subunits. DUOX2 is one of the most conserved NOX isoforms and in line with this, DUOXA2 is well conserved (39%). However, for the highly conserved NOX3, the situation is different: its obligatory subunit NOXO1 does not show any regions of high conservation. DUOX1 shows a moderate degree of conservation similarly to DUOXA1. However, for the rest of the NOX system, such correlations do not hold. NOX1 shows some degree of conservation, but its subunits NOXO1 and NOXA1 are not conserved at all. NOX2, which only shows a very small area of conservation (11 amino acids), but its subunit NCF2 is the most highly conserved (73%), and its other two subunits NCF1 and NCF4 also show some degree of conservation (11% and 31%, respectively). CYBA, a common transmembrane subunit of NOX1-4, also shows a moderate degree of conservation.

The massive difference between the conservation of the two DUOX enzymes is puzzling. None of the two enzyme systems seems to be dispensable as witnessed by the presence of the 2 isoforms in all mammalian species investigated in our study. Yet, the amino acid sequence of DUOX2/DUOXA2 is highly conserved, while DUOX1 only shows a moderate degree of conservation and DUOXA1 is less well conserved than DUOXA2.

The absence of DUOXA1 and DUOXA2 in the giant panda is of great interest. Indeed, thyroid hormone levels are extremely low in this species compared to their mammalian norm. DUOX2 is essential for thyroid hormone synthesis and this specific hypothyroidism has been attributed to the presence of a unique mutation in the DUOX2 gene of the giant panda [24,25]. As DUOXA2 is essential for DUOX2 function [26]-and cannot be complemented by DUOXA1, our findings bring a so far unexplored possibility for the low thyroid hormone levels found in the giant panda.

The reasons for the high conservation of DUOX2 remain enigmatic. With respect to the dehydrogenase domain, there are some indications that the catalytic activity of DUOX2 might not be limited to NADPH, but that it might also metabolize NAADPH [27], which obviously would put a restraint on the sequence flexibility. Within the highly conserved regions of DUOX2, there are also functionally important cysteines: Cysteines 568 and 582 form intermolecular disulfide bonds with cysteines 167 and 233 of DUOXA2, which play a significant role in DUOXA2 stability and function. Cysteine 1162 forms an intramolecular disulfide bond with cysteine 124 and is crucial for DUOX2 function [28].

Within the relatively small area of DUOX1 conservation, there is one interesting lead: the conserved region within transmembrane domain 7 is precisely within the stretch that forms an interface with DUOXA1. Note however that other DUOX1 domains that interface with DUOXA1 are not conserved.

The NOX2 subunit NCF2 exhibits a very high level of conservation. The conserved amino acids are distributed across domains that interact with Rac, NOX2, and NCF1. The tetratricopeptide domain of NCF2, which interacts with Rac, is almost completely conserved across mammals. The AD and the second SH3 domains, which respectively interact with NOX2 and the proline-rich repeats of NCF1, are also strongly conserved. Regarding NCF1, almost all the conserved amino acids are in the first SH3 domain. This domain is crucial for NCF1's interaction with CYBA, and it plays a critical role in activating the enzyme and recruiting other subunits. In NCF4, the N-terminal and PX domains, which specifically interact with phosphatidylinositol 3-phosphate and play a crucial role in stabilizing the NOX complex, are conserved. Additionally, conserved amino acids are observed in most of the SH3 domain, which interacts with the proline-rich region of NCF1.

In CYBA, there is a stretch of 9 aa within the transmembrane domains of the protein. This stretch is close to a region that is supposed to be important for NOX2 maturation [29,30]. It is not clear whether this area is also important for the maturation of the CYBA-interacting NOX isoforms (NOX1, NOX3, and NOX4). Interestingly, the proline-rich domain of CYBA (amino acids 151–160), which binds to the SH3 domain of NCF1 and NOXO1, is not conserved.

Taken together, protein-protein interactions may in part explain the high degree of conservation of certain stretches within NOX isoforms and subunits, however, they certainly do not explain the entire picture. Other protein functionalities such as intramolecular interactions might be important. Protein architecture and stability is most likely another relevant factor for high amino acid conservation.

Loss of function mutations in the genes coding for NOX2, NCF1, NCF2, and NCF4, NOX3, and DUOX2 cause severe phenotypes in humans and in animal models. This concurs with the observation that none of these NOX are lost during mammalian evolution and that these enzymes show a high degree of amino acid conservation (with the notable exception of NOX2). On the other extreme, there are NOXA1, and NOX5 for which no loss of function mutations with a detectable phenotype are known, and these enzymes or subunits can be lost during evolution and show the least amount of amino acid conservation. However, such correlations cannot be made for the remaining isoforms.

NOX5 is puzzling with respect to evolutionary aspects. NOX5 is the ancestral NOX isoform during eukaryotic evolution; it has multiplicated in plants, where up to 10 NOX5-like genes with distinct physiological functions are expressed [16]. Yet, its degree of conservation is the lowest among mammalian NOX isoforms, and it was even lost several times during mammalian evolution (subfraction of rodents, lagomorphs, and bats). The high degree of amino acid variability in NOX5 might be due to the resilience of NOX5 to amino acid changes, indicating a particular robustness of the protein structure. Additionally, NOX5 is the only NOX, that functions without the need for subunits, which might give the enzyme a greater liberty for the evolution of its amino acid sequences.

## 5 Conclusion

In this study, we conducted a comprehensive investigation of NOX isoforms and their subunits in 164 mammalian species available in the ortholog database, with a focus on their presence in the species with available sequence and their conservation during evolution. Our results reveal that, in all evaluated mammalian species, NOX2, NOX3, NOX4, as well as DUOX1 and DUOX2 are present. However, some mammalian species lost NOX1, while others lost NOX5. The observation that NOX1 is absent in Afrotheria and Monotremata is entirely novel and may lead to a better understanding of the physiological role of NOX1. Prior to our study, it was already known that NOX5 is absent in mice and rats, and it had been assumed that this might apply to all rodents. We showed that in the squirrel and the marmot from the squirrel families of rodents, NOX5 is present. Investigation of rodent species with and without NOX5 will be of great scientific interest as it will shed light on our understanding of the function of NOX5, particularly in spermatogenesis.

A surprising finding of our study is the fact that amino acid conservation is highly variable between different NOX isoforms. For example, the highly conserved “MFLYLCERLVR” sequence in the hinge region between the transmembrane domain and the dehydrogenase domain of NOX2 is absent in all other NOX isoforms and that there is even no high degree of conservation of the hinge region of the NOX isoforms NOX1, NOX4, NOX5, and DUOX1. Note that these highly conserved, isoform-defining sequences have not been described before and hence, our evolutionary analysis provides material for the generation of new hypothesis on molecular function of NOX isoforms.

The comparison of amino acid sequences of NOX isoforms revealed residues that are highly conserved among all mammalian species, which have not been identified thus far. The highest level of conservation was observed in NOX3 and DUOX2, as well as the NCF2 subunit. Importantly, none of these components have been lost in mammalian species during evolution. Since mutations in the genes encoding NCF2, NOX3, and DUOX2 cause severe phenotypes in humans and animal models, this finding highlights a correlation between a high level of conservation and the essentiality of these enzymes. The only exception is NOX2, which, despite causing severe phenotypic effects when mutated, is not as well conserved. Therefore, further studies are needed to discover the role of conserved residues in the functioning of these enzymes.

## Authors contributions

K-H. K. designed the experiments; K-H. K. and B.N. performed the experiments; K-H. K., B.N. and V. J. wrote the manuscript and prepared the figures.

## Funding

This study was granted by 10.13039/100000001Swiss National Science Foundation to Karl-Heinz Krause (Grant 31003A-179478).

## Declaration of competing interest

All authors have no competing interests to declare.

## Figures and Tables

**figs1:**
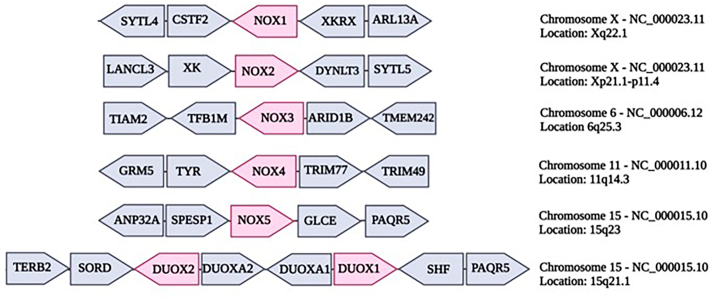


**figs2:**
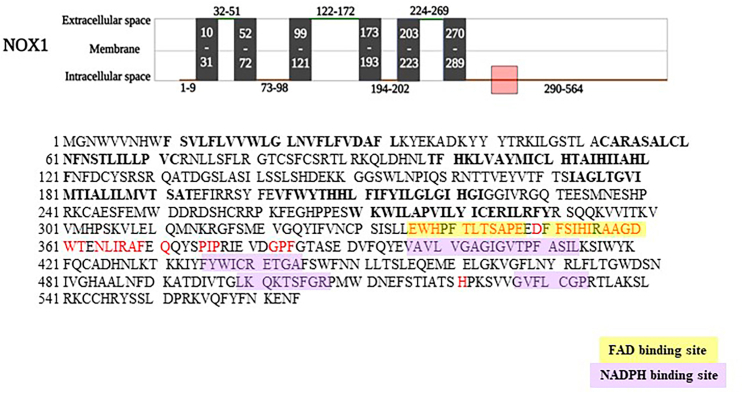


**figs3:**
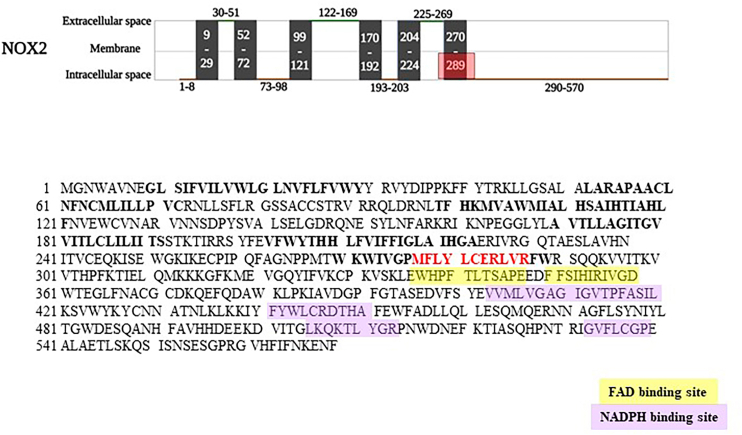


**figs4:**
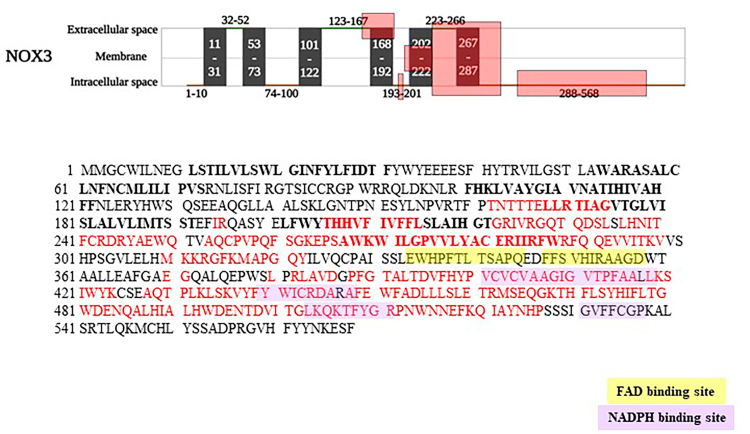


**figs5:**
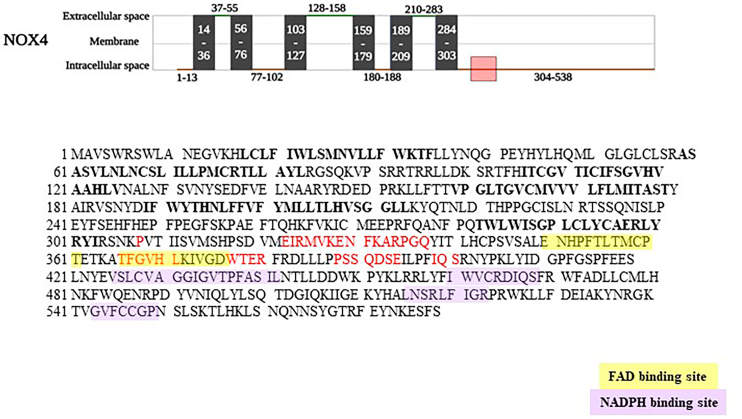


**figs6:**
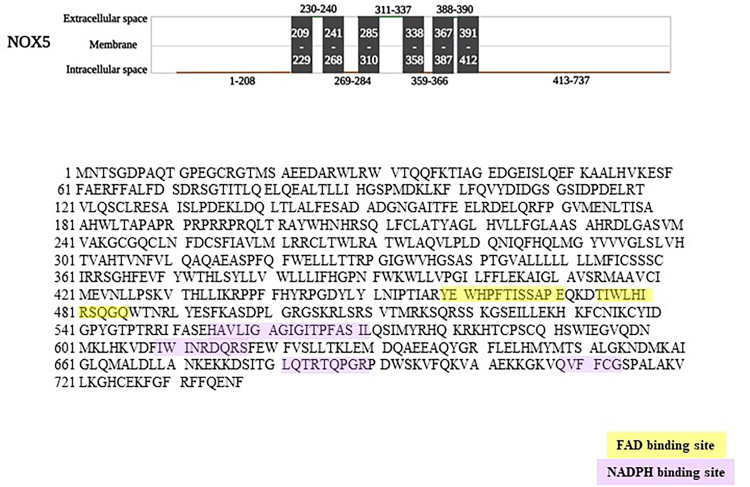


**figs7:**
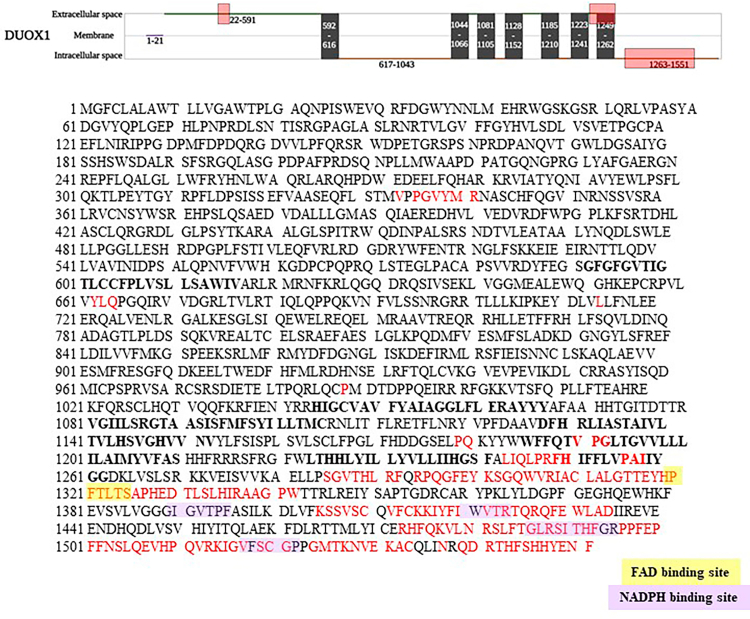


**figs8:**
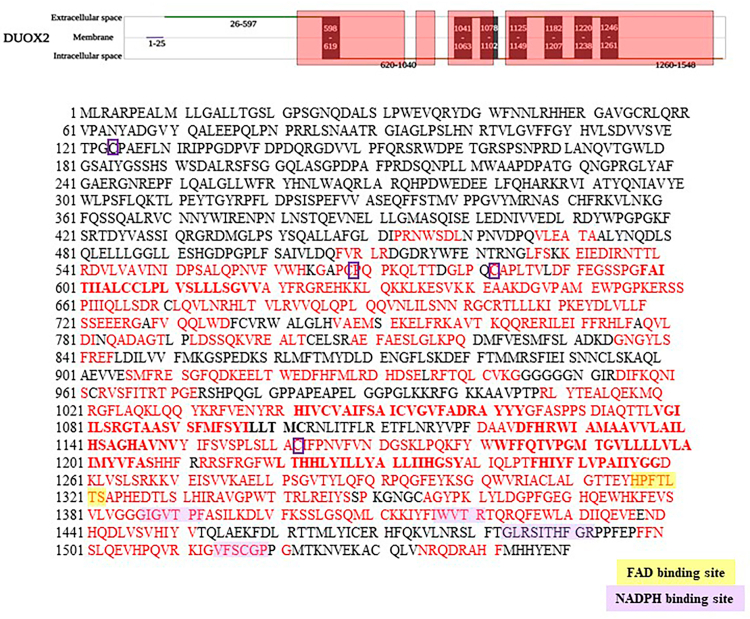


**figs9:**
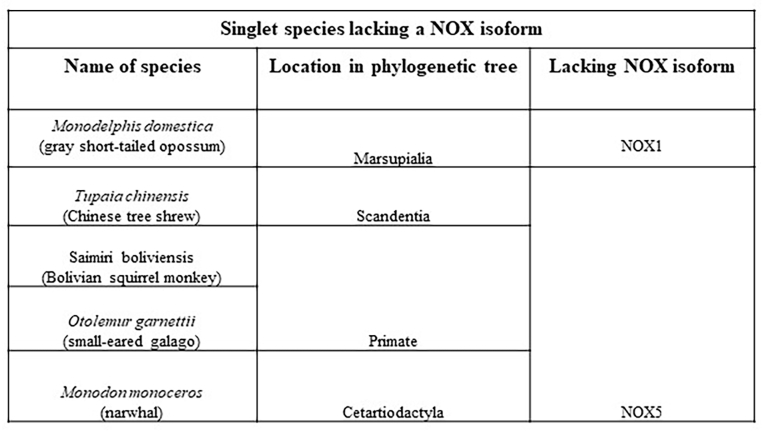


## Data Availability

We have made the data utilized in this research available through an Excel file, included as supplementary data. Additionally, the links to all databases used are provided in the Methods section.
