# Supplementary material for: NOX family NADPH oxidases in mammals: Evolutionary conservation and isoform-defining sequences
Source: Redox Biol. 2023 Aug 12;66:102851. doi: 10.1016/j.redox.2023.102851 (PMC10458973; doi:10.1016/j.redox.2023.102851)
Supplement: Multimedia component 1 [file mmc1.docx]

Supplementary figures’ legend

Supplementary Table 1. Singlet species without a specific NOX isoform for which there is no evolutionary related species.

Supplementary Figure 1. Neighboring genes of each NOX isoform. When a given NOX isoform was absent in orthologs database, the neighboring genes were used to inspect the respective genomic region.

Supplementary Figure 2. Human NOX1 structure and sequence, red indicates highly conserved amino acids among mammals. Transmembrane domains are shown in bold. Yellow & violet highlight FAD & NADPH binding sites, respectively.

Supplementary Figure 3. Human NOX2 structure and sequence, red indicates conserved amino acids among mammals. Transmembrane domains are shown in bold. Yellow & violet highlight FAD & NADP binding sites, respectively.

Supplementary Figure 4. Human NOX3 structure and sequence, red indicates conserved amino acids among mammals. Transmembrane domains are shown in bold. Yellow & violet highlight FAD & NADPH binding sites, respectively.

Supplementary Figure 5. Human NOX4 structure and sequence, red indicates conserved amino acids among mammals. Transmembrane domains are shown in bold. Yellow & violet highlight FAD & NADPH binding sites, respectively.

Supplementary Figure 6. Human NOX5 structure and sequence. Transmembrane domains are shown in bold. Yellow & violet highlight FAD & NADP binding sites, respectively.

Supplementary Figure 7. Human DUOX1 structure and sequence, red indicates conserved amino acids among mammals. Transmembrane domains are shown in bold. Yellow & violet highlight FAD & NADPH binding sites, respectively.

Supplementary Figure 8. Human DUOX2 structure and sequence, red indicates conserved amino acids among mammals. Transmembrane domains are shown in bold. Yellow & violet highlight FAD & NADPH binding sites, respectively.
